# Short-course versus standard- or longer-course oral antibiotic therapy for pneumonia and lower respiratory tract infection in children: a systematic review and meta-analysis of randomized controlled trials

**DOI:** 10.3389/fphar.2026.1877367

**Published:** 2026-10-14

**Authors:** Lian Zhou, Shanshan Wang, Yuyi Tang, Jinlin Chen, Xingfeng Cheng

**Affiliations:** 1 Emergency and Critical Care Medicine Center, Wuhan Children's Hospital (Wuhan Maternal and Child Healthcare Hospital), Tongji Medical College, Huazhong University of Science and Technology, Wuhan, China; 2 Outpatient Department, Wuhan Children's Hospital (Wuhan Maternal and Child Healthcare Hospital), Tongji Medical College, Huazhong University of Science and Technology, Wuhan, China; 3 The First School of Clinical Medicine, Nanjing Medical University, Nanjing, China; 4 Outpatient Office, Wuhan Children's Hospital (Wuhan Maternal and Child Healthcare Hospital), Tongji Medical College, Huazhong University of Science and Technology, Wuhan, China

**Keywords:** antimicrobial stewardship, pediatric infections, short-course antibiotics, systematic review, treatment failure

## Abstract

**Background:**

Reducing unnecessary antibiotic exposure is a major priority in pediatric antimicrobial stewardship. The comparative effectiveness of short-course versus standard- or longer-course oral antibiotic therapy for uncomplicated bacterial infections in children remains uncertain.

**Objective:**

To evaluate whether short-course oral antibiotic therapy increases clinical treatment failure compared with standard- or longer-course therapy in children with uncomplicated bacterial infections, with a primary focus on same-antibiotic pediatric pneumonia/lower respiratory tract infection (LRTI) trials.

**Methods:**

We conducted a systematic review and meta-analysis of randomized controlled trials. MEDLINE/PubMed, Embase, Web of Science, and CENTRAL were searched, supplemented by citation searching and trial registry review. Eligible studies enrolled children aged 0–18 years with uncomplicated bacterial infections treated with oral antibiotics in outpatient or ambulatory settings. The primary outcome was clinical treatment failure; broader pneumonia/LRTI and all-infection analyses were treated as sensitivity or exploratory analyses. Random-effects meta-analysis used risk ratios (RRs) and risk differences (RDs) with 95% confidence intervals (CIs). Risk of bias was assessed with the Cochrane risk-of-bias tool. The review was registered in PROSPERO (CRD420261339263).

**Results:**

Fourteen randomized trials were included; nine same-antibiotic pneumonia/LRTI comparisons contributed to the primary pooled analysis. Short-course therapy was not associated with increased treatment failure (RR, 1.01; 95% CI, 0.92–1.11; I^2^ = 0.0%), and the absolute-effect analysis showed no meaningful difference (RD, 0.001; 95% CI, −0.010 to 0.013; I^2^ = 10.0%). A broad pneumonia/LRTI sensitivity analysis including different-antibiotic comparisons was also non-significant (RR, 0.94; 95% CI, 0.66–1.33) but heterogeneous. The all-infection exploratory analysis was similarly non-significant (RR, 0.89; 95% CI, 0.63–1.25) and interpreted cautiously given clinical heterogeneity. Of the 14 trials, 5 were at low risk of bias, 2 had some concerns, and 7 were at high risk.

**Conclusion:**

In same-antibiotic pediatric pneumonia/LRTI trials, short-course therapy was not associated with increased clinical treatment failure compared with standard- or longer-course therapy. Broader analyses across different antibiotic strategies and infection syndromes did not materially change this interpretation but showed substantial heterogeneity. These findings support cautious, infection-specific antibiotic duration reduction rather than broad generalization across all uncomplicated pediatric bacterial infections.

**Systematic Review Registration:**

https://www.crd.york.ac.uk/PROSPERO/view/CRD420261339263, identifier CRD420261339263.

## Introduction

Antimicrobial resistance is a major global health threat, and reducing unnecessary antibiotic exposure is a central goal of antimicrobial stewardship (Barlam et al., 2016). In pediatric practice, common uncomplicated bacterial infections remain among the most frequent indications for antibiotic prescribing in outpatient and ambulatory settings (Fleming-Dutra et al., 2016; Hersh et al., 2013). Although antibiotics are often necessary, excessive duration of therapy may increase the risk of adverse drug effects, alter the developing microbiome, increase healthcare costs, and contribute to selective pressure for antimicrobial resistance without improving clinical outcomes (Barlam et al., 2016; Hersh et al., 2013; Langdon et al., 2016).

Historically, antibiotic treatment durations have often been based more on convention than on high-quality comparative evidence (Langdon et al., 2016; Spellberg, 2016). The long-standing assumption that longer treatment is inherently safer or more effective has increasingly been challenged, particularly for infections in which early clinical response is rapid and bacterial burden is low (Langdon et al., 2016; Spellberg, 2016). This issue is especially relevant in children, in whom oral antibiotics are commonly prescribed for community-acquired pneumonia, acute otitis media, and urinary tract infection. These conditions account for a substantial proportion of pediatric antibiotic exposure and therefore represent key targets for stewardship-focused optimization (Fleming-Dutra et al., 2016; Hersh et al., 2013; Hersh et al., 2011; Llewelyn et al., 2017).

Over the past decade, randomized trials have begun to evaluate whether shorter oral antibiotic regimens can achieve clinical outcomes comparable to standard-duration therapy in selected pediatric infections (Lieberthal et al., 2013; Haider et al., 2008). For pediatric community-acquired pneumonia, recent trials have suggested that shorter-course treatment may be non-inferior to longer regimens in carefully selected outpatient populations (Lieberthal et al., 2013; Haider et al., 2008). At the same time, evidence from other infections, including acute otitis media and urinary tract infection, remains more heterogeneous because of differences in trial design, comparator strategies, outcome definitions, and follow-up time points (Llewelyn et al., 2017; Benkendorff et al., 2026). Earlier reviews have often focused on single syndromes rather than providing an integrated synthesis of randomized evidence across common uncomplicated pediatric bacterial infections (Daws et al., 2017).

This creates an important clinical and antimicrobial-stewardship question: among children with common uncomplicated bacterial infections requiring oral antibiotic therapy, does short-course oral antibiotic treatment, compared with standard- or longer-course oral antibiotic treatment, achieve similar treatment success without increasing clinical treatment failure? Accordingly, we conducted a systematic review and meta-analysis of randomized controlled trials to evaluate whether shorter oral antibiotic regimens are associated with increased treatment failure and to inform antimicrobial stewardship in pediatric practice.

## Methods

### Study design and registration

This systematic review and meta-analysis was registered with PROSPERO (CRD420261339263) and conducted in accordance with PRISMA 2020 (Page et al., 2021).

### Eligibility criteria

We included randomized controlled trials enrolling children and adolescents aged 0–18 years with uncomplicated bacterial infections treated with oral antibiotics. Eligible clinical condition included uncomplicated community-acquired pneumonia or lower respiratory tract infection (LRTI), streptococcal pharyngitis or tonsillitis, uncomplicated urinary tract infection, acute otitis media, acute bacterial sinusitis, and uncomplicated skin and soft tissue infection, as defined by the diagnostic criteria used in each trial.

Studies were eligible if they compared a shorter oral antibiotic course with a standard- or longer-course oral antibiotic regimen for treatment of the same uncomplicated infection. Short-course therapy was defined according to the regimen evaluated within each randomized trial rather than by a single universal numerical threshold. This approach was used because the clinical meaning of “short-course” differs by infection syndrome and comparator duration; for example, 5 days may be the shorter course in a 5-versus-10-day comparison but the longer course in a 3-versus-5-day comparison. Studies were excluded if they enrolled predominantly adult populations without extractable pediatric data; evaluated severe, invasive, chronic, recurrent, hospital-acquired, or healthcare-associated infections; focused on prophylaxis rather than treatment; involved predominantly parenteral antibiotic therapy; enrolled immunocompromised populations; or did not provide a clear short-course versus standard- or longer-course oral antibiotic comparison.

### Information sources and search strategy

We searched the Cochrane Central Register of Controlled Trials (CENTRAL), Embase via Ovid, MEDLINE/PubMed, and Web of Science from database inception to 1 May 2026. The search was limited to published English-language studies, consistent with the PROSPERO protocol. No publication-date restrictions were applied. Two trial registries ClinicalTrials.gov and the WHO International Clinical Trials Registry Platform (ICTRP) were also searched for completed, ongoing, and unpublished trials, and backward and forward citation searching (screening reference lists of included trials and relevant reviews, and forward-citation tracking in Web of Science and Google Scholar) was performed. Web of Science was searched in addition to the databases registered in the PROSPERO record. Because PubMed and MEDLINE overlap substantially, PubMed/MEDLINE was treated as one biomedical search source for reporting purposes. PubMed was used to identify MEDLINE-indexed records and records not yet fully indexed in MEDLINE, and all retrieved records were deduplicated before screening. Two reviewers (LZ and SW) independently screened all titles and abstracts against the eligibility criteria and then independently assessed the full text of every potentially eligible record; disagreements at either stage were resolved by discussion, with adjudication by a senior reviewer (JC or XC) where needed, and reasons for full-text exclusion were recorded. Embase, CENTRAL, and Web of Science were searched as additional bibliographic sources. The search strategy combined controlled vocabulary and free-text terms for children, oral antibiotic therapy, short-course or reduced-duration therapy, standard- or longer-course therapy, randomized trials, and common uncomplicated pediatric bacterial infections, including pneumonia/LRTI, urinary tract infection, pharyngitis/tonsillitis, acute otitis media, sinusitis, and skin and soft tissue infection. The complete database-specific search strategies are provided in Supplementary Appendix S1.

### Data extraction and outcomes

Two reviewers independently extracted data using a standardized data-extraction form. Extracted variables included first author, publication year, country, study design, setting, sample size, age range, diagnostic method, infection syndrome, antibiotic regimen, dose, frequency, treatment duration in the intervention and comparator arms, follow-up time point, outcome definition, and event data. For dichotomous outcomes, the number of events and total number of participants were extracted separately for the short-course and standard- or longer-course groups. When a study reported more than one eligible randomized duration comparison, each comparison was extracted separately while preserving the study identifier.

The primary outcome was clinical treatment failure, defined as persistence, non-resolution, worsening of infection-related signs or symptoms, relapse requiring additional treatment, or need for antibiotic retreatment at the test-of-cure visit or nearest comparable follow-up time point reported in each trial. Because outcome labels differed across studies, clinically comparable unfavorable outcomes were harmonized as treatment failure.

### Quality assessment

Risk of bias was assessed independently by two reviewers using the Cochrane Risk of Bias 2 (RoB 2) tool. Disagreements were resolved by discussion (Sterne et al., 2019). The certainty of evidence for the primary outcome was assessed using the Grading of Recommendations Assessment, Development and Evaluation (GRADE) approach.

### Data synthesis and statistical analysis

All meta-analyses were conducted in R using the “meta” package. The risk ratio (RR) was used as the primary relative effect measure, and the risk difference (RD) was used as the absolute effect measure. Effect estimates were reported with 95% confidence intervals (CIs). Trial-level data were pooled using the inverse-variance method under a DerSimonian–Laird random-effects model, chosen because clinical and methodological heterogeneity was expected across trials; 95% confidence intervals were derived from the normal approximation, and the Hartung–Knapp adjustment was not applied. For ratio measures, a continuity correction of 0.5 was applied to comparisons with a zero event cell, and comparisons with zero events in both arms were excluded from risk-ratio pooling because the ratio is undefined. For the factorial CAP-IT trial, only the treatment-duration comparison was analyzed; for trials contributing more than one duration stratum, each short-versus-comparator stratum was entered as a separate comparison, and shared control arms were not double-counted. Cluster-randomized trials (Awasthi 2008; Sadruddin 2019) contributed only to the broad sensitivity and exploratory analyses and were entered using the aggregate event counts reported by the investigators. Event data corresponded to each trial’s outcome-evaluable (modified intention-to-treat) population. Analyses were performed in R version 4.6.0 (meta package, version 8.3–0). Between-study heterogeneity was assessed using the I^2^ statistic, τ^2^, and Cochran’s Q test. I^2^ values were reported as percentages.

The main inference was based on the primary same-antibiotic pneumonia/LRTI treatment-failure analysis. Sensitivity analyses included a broad pneumonia/LRTI analysis incorporating different-antibiotic comparisons and leave-one-out analyses of the primary dataset. The all-infection analysis was considered exploratory and was not used as the main basis for inference because of clinical heterogeneity. Small-study effects were assessed using funnel plots and Egger’s regression test. Funnel plots and Egger’s tests were reported for the broad pneumonia/LRTI sensitivity analysis and the all-infection exploratory analysis.

## Results

### Study selection

The study-selection process is summarized in Figure 1. The search identified 1,352 records from databases and other sources. After removal of 1,083 duplicate records, 269 records were screened, of which 212 were excluded. Fifty-seven full-text reports were assessed for eligibility. Fourteen randomized trials were included in the systematic review. The trials contributing to each pooled analysis are listed in Supplementary Table S4, and the reconciliation between the numbers randomised in Table 1 and the numbers contributing to the primary pooled analysis is given in Supplementary Table S5.

**FIGURE 1:**
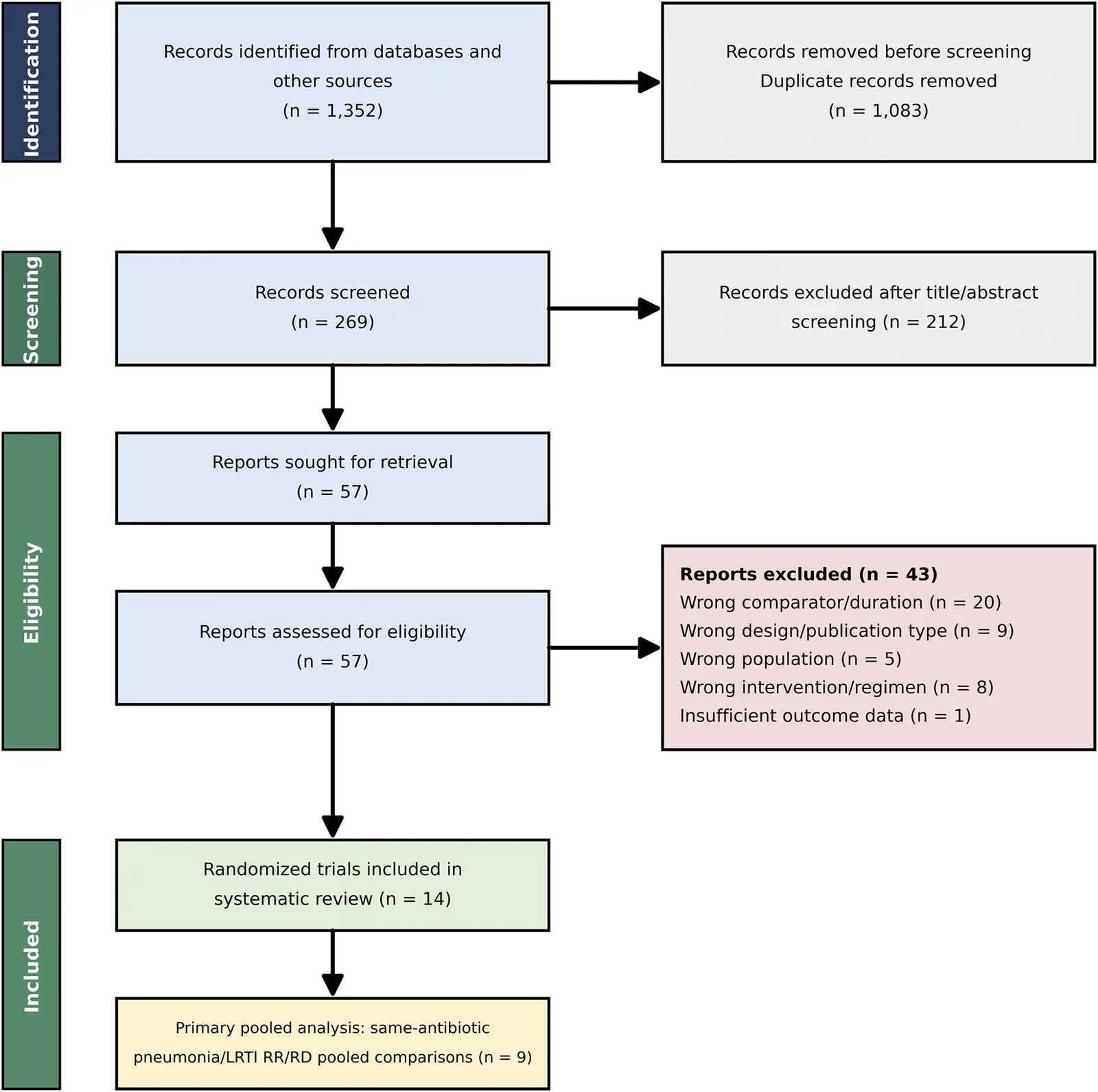
PRISMA 2020 flow diagram of study selection (adapted from Page et al., 2021; CC BY 4.0; BMJ 372:n71).

**TABLE 1:** Characteristics of included randomized clinical trials evaluating short-course versus standard- or longer-course antibiotic therapy in children.

Study	Country	Study design	Sample size	Age	Method of diagnosis	Classification/condition	Antibiotics	Dose	Frequency	Duration, intervention vs. control, d
SCOUT-CAP, 2022 (Williams et al., 2022)	US	RCT	385	6–71 months	Clinical	Non-severe uncomplicated community-acquired pneumonia	Amoxicillin^a^	80–100 mg/kg/d	Twice daily	5 vs. 10
CAP-IT, 2021 (Bielicki et al., 2021)	UK and Ireland	RCT, factorial	824	6–59 months	Clinical	Chest-indrawing or fast-breathing pneumonia	Amoxicillin	Standard dose: 35–50 mg/kg/d; high dose: 70–90 mg/kg/d	Twice daily	3 vs. 7
SAFER, 2021 (Pernica et al., 2021)	Canada	RCT	281	6 months–10 years	Radiographic	Chest-indrawing or fast-breathing pneumonia	Amoxicillin	75–100 mg/kg/d	3 times daily	5 vs. 10
Ginsburg et al., 2020 (Ginsburg et al., 2020)	Malawi	RCT	3,000	2–59 months	Clinical	Chest-indrawing pneumonia	Amoxicillin	2–11 months: 500 mg/d; 12–35 months: 1,000 mg/d; 36–59 months: 1,500 mg/d	Twice daily	3 vs. 5
(Greenberg et al., 2014)	Israel	RCT, sequential	140	6–59 months	Radiographic	alveolar pneumonia	Amoxicillin	80 mg/kg/d	3 times daily	3 vs. 10
ISCAP, 2004 (Agarwal et al., 2004)	India	RCT	2,188	2–59 months	Clinical	Fast-breathing pneumonia	Amoxicillin	31–54 mg/kg/d	3 times daily	3 vs. 5
Kartasasmita et al., 2004 (World Health Organization, 2004)	Indonesia and Bangladesh	RCT (WHO report)	2,024	2–59 months	NR	NR	Cotrimoxazole	NR	NR	3 vs. 5
MASCOT, 2002 (Pakistan Multicentre Amoxycillin Short Course Therapy MASCOT Pneumonia Study Group, 2002)	Pakistan	RCT	2,000	2–59 months	Clinical or radiographic	Fast-breathing pneumonia	Amoxicillin	45 mg/kg/d	3 times daily	3 vs. 5
(Lupisan et al., 1999)	Philippines	RCT	303	2–59 months	Clinical	Fast-breathing pneumonia	Cotrimoxazole	≥12 months: 80 mg/dose; 2–12 months: 40 mg/dose	Twice daily	3 vs. 5
(Sadruddin et al., 2019)	Pakistan	Cluster RCT	4,984	2–59 months	Clinical	Fast-breathing pneumonia, community-managed	Amoxicillin vs. cotrimoxazole	Amoxicillin: 50 mg/kg/d; cotrimoxazole: 40 mg SMX/8 mg TMP/kg/d	Twice daily	3 vs. 5
(Li et al., 2019)	China	RCT	256	2–12 years	Rapid strep test	Group A *Streptococcus tonsillitis*	Azithromycin or cefaclor	Azithromycin: 10 mg/kg/d; cefaclor: 20 mg/kg/d	Once daily for azithromycin; 3 times daily for cefaclor	3 vs. 10; 5 vs. 10
STOP, 2024 (Montini et al., 2024)	Italy	RCT	142	3 months–5 years	Clinical + urine culture	Febrile urinary tract infection	Amoxicillin-clavulanate	50 + 12.5 mg/kg/d	3 times daily	5 vs. 10
(Roord et al., 1996)	The Netherlands	Prospective open randomized study	85	2–16 years	NR	Community-acquired acute lower respiratory tract infection	Azithromycin vs. erythromycin	NR	NR	3 vs. 10
(Awasthi et al., 2008)	India	RCT	2009	2–59 months	Clinical	Nonsevere pneumonia with or without wheez	Amoxicillin vs. cotrimoxazole	Amoxicillin: 50 mg/kg/d Cotrimoxazole: 40 mg/kg/d	2 time daily	3 vs. 5

^a^Dose range as reported in the original trial publication.

Abbreviations: CAP, community-acquired pneumonia; LRTI, lower respiratory tract infection; mo, months; NR, not reported; RCT, randomized controlled trial; SMX, sulfamethoxazole; TMP, trimethoprim; UK, united kingdom; US, united states; y, years. RCT (WHO, report) denotes a randomized controlled trial whose results are reported in a World Health Organization document (WHO/FCH/CAH/04.2) rather than in a separate peer-reviewed journal article.

### Study characteristics

The main characteristics of the included trials are shown in Table 1. Most trials evaluated children with uncomplicated community-acquired pneumonia, fast-breathing pneumonia, chest-indrawing pneumonia, or other non-severe pneumonia/LRTI syndromes. One trial evaluated febrile urinary tract infection, and two ancillary comparisons involved tonsillitis/pharyngotonsillitis or acute lower respiratory tract infection treated with different antibiotic regimens.

The studies included were conducted in both high-income countries and low- and middle-income countries. Treatment-duration comparisons included 3 versus 5 days, 3 versus 7 days, 3 versus 10 days, and 5 versus 10 days. The most frequently evaluated antibiotic was amoxicillin. Some trials, including Awasthi 2008, Sadruddin 2019, Li 2019, and Roord 1996, compared different antibiotic classes as well as different treatment durations; these were therefore not considered pure same-antibiotic duration comparisons in the primary analysis.

### Primary outcome: treatment failure

primary analysis restricted to same-antibiotic pneumonia/LRTI duration-comparison trials, short-course therapy was not associated with an increased risk of treatment failure compared with standard- or longer-course therapy. The pooled relative effect was close to the null, with a risk ratio (RR) of 1.01 (95% CI, 0.92 to 1.11; I^2^ = 0.0%) (Figure 2). The corresponding absolute-effect analysis also showed no meaningful difference between groups. The pooled risk difference (RD) was 0.001 (95% CI, −0.010 to 0.013; I^2^ = 10.0%) (Supplementary Figure S1). The certainty of evidence for the primary outcome was moderate (GRADE), downgraded one level for risk of bias (Supplementary Table S3).

**FIGURE 2:**
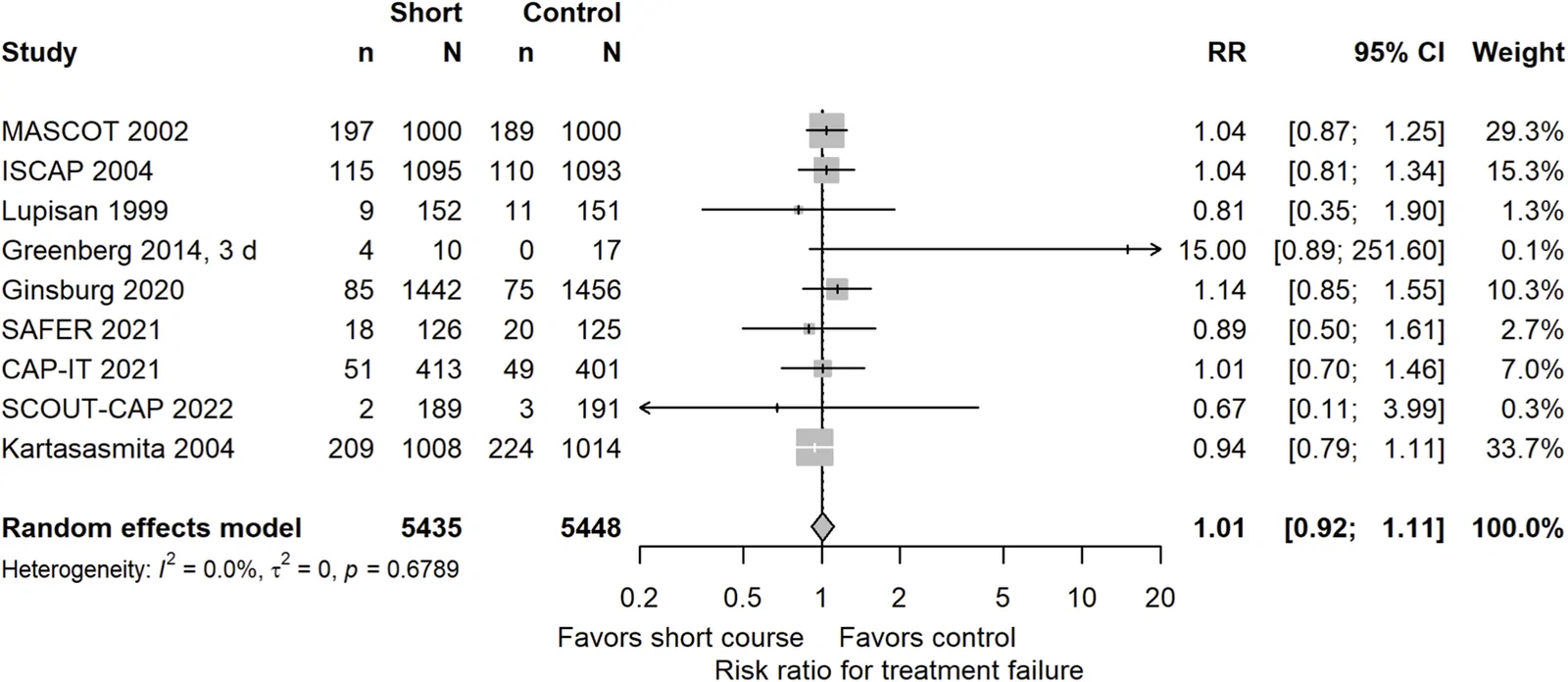
Primary treatment-failure meta-analysis in same-antibiotic pediatric pneumonia/LRTI trials. RR indicates risk ratio; CI, confidence interval. Short indicates short-course therapy; Control indicates standard- or longer-course therapy.

### Sensitivity and exploratory analyses

Leave-one-out sensitivity analyses of the primary outcome showed stable findings. Sequential exclusion of individual comparisons produced pooled RDs close to the null, indicating that indicate that the primary result was not driven by any single same-antibiotic pneumonia/LRTI comparison. The details are shown in Supplementary Table S1.

An exploratory all-infection analysis remained statistically non-significant (RR, 0.89; 95% CI, 0.63–1.25) (Supplementary Table S2), but heterogeneity was substantial (I^2^ = 92.8%; τ^2^ = 0.27; Cochran’s Q = 165.8, df = 12; P < 0.001). Because this analysis combined clinically different infection syndromes and treatment strategies, it was interpreted as exploratory only and was not used as the main basis for inference.

Broad pneumonia/LRTI sensitivity analysis. In this prespecified sensitivity analysis, the three trials that differed in antibiotic class as well as duration (Sadruddin 2019; Awasthi 2008; and Roord 1996) were added to the nine primary comparisons, giving 12 comparisons (short-course n = 15,626; comparator n = 13,013). The pooled risk ratio for treatment failure was 0.94 (95% CI, 0.66–1.33) with substantial heterogeneity (I^2^ = 93.2%; τ^2^ = 0.27; Cochran’s Q = 162.5, df = 11; P < 0.001), and the corresponding risk difference was −0.002 (95% CI, −0.029 to 0.025; I^2^ = 90.1%). The forest plot for this analysis is shown in Supplementary Figure S6. Because this analysis incorporated different-antibiotic comparisons and showed high heterogeneity, its result is interpreted as exploratory rather than confirmatory.

Funnel plots for the broad pneumonia/LRTI sensitivity analysis and the all-infection exploratory analysis did not show clear evidence of asymmetry (Supplementary Figures S2 and S3). Egger’s test was not statistically significant in the broad pneumonia/LRTI sensitivity analysis (P = 0.25) or the all-infection exploratory analysis (P = 0.36). These findings should be interpreted cautiously because the number of comparisons remained limited.

### Risk of bias assessment

Risk-of-bias assessments are summarized in Supplementary Figures S4 and S5. Among the 14 included randomized trials, 5 were judged to be at low overall risk of bias, 2 had some concerns, and 7 were judged to be at high risk of bias. The most common concerns were lack of blinding in open-label trials, unclear allocation concealment in older studies, incomplete outcome data in selected trials, and potential other bias related to cluster randomization or simultaneous differences in antibiotic class and treatment duration. The GRADE analysis can be seen in Supplementary Table S3.

## Discussion

In this systematic review and meta-analysis of randomized clinical trials, short-course oral antibiotic therapy was not associated with a statistically detectable increase in treatment failure relative to standard- or longer-course therapy for children with common uncomplicated bacterial infections. Across 9 same-antibiotic pneumonia/lower respiratory tract infection comparisons, the pooled relative and absolute effects were close to the null, with an RR of 1.01 (95% CI, 0.92 to 1.11; I^2^ = 0%) and an RD of 0.001 (95% CI, −0.010–0.013). These findings suggest that, within the randomized evidence included in this review, shorter antibiotic courses were not associated with a statistically detectable increase in treatment failure compared with standard- or longer-course regimens. Because no non-inferiority or equivalence margin was prespecified, this indicates the absence of a detectable difference rather than formal evidence of equivalence (Williams et al., 2022; Bielicki et al., 2021; Pernica et al., 2021; Ginsburg et al., 2020; Greenberg et al., 2014; Agarwal et al., 2004; World Health Organization, 2004; Pakistan Multicentre Amoxycillin Short Course Therapy MASCOT Pneumonia Study Group, 2002; Lupisan et al., 1999).

These findings are clinically relevant because reducing unnecessary antibiotic exposure is a central goal of pediatric antimicrobial stewardship (Barlam et al., 2016; Fleming-Dutra et al., 2016; Hersh et al., 2013; Awasthi et al., 2008; Gao et al., 2023). Longer antibiotic courses may increase adverse events, disrupt the developing microbiome, increase treatment burden and cost, and contribute to selective pressure for antimicrobial resistance (Barlam et al., 2016; Langdon et al., 2016; Spellberg, 2016; Llewelyn et al., 2017). However, the present findings should not be interpreted as evidence that shorter therapy is superior to longer therapy. Rather, they support the interpretation that shorter regimens can be clinically comparable to standard- or longer-course regimens in selected pediatric infections when used in appropriate populations and duration comparisons.

A major consideration is the clinical heterogeneity of the evidence included. Trials differed by infection syndrome, diagnostic criteria, antibiotic regimen, dose, treatment-duration contrast, healthcare setting, and outcome definition (Williams et al., 2022; Bielicki et al., 2021; Pernica et al., 2021; Ginsburg et al., 2020; Greenberg et al., 2014; Agarwal et al., 2004; World Health Organization, 2004; Pakistan Multicentre Amoxycillin Short Course Therapy MASCOT Pneumonia Study Group, 2002; Lupisan et al., 1999). Most evidence came from pneumonia trials, including children with non-severe community-acquired pneumonia, fast-breathing pneumonia, or chest-indrawing pneumonia (Williams et al., 2022; Bielicki et al., 2021; Pernica et al., 2021; Ginsburg et al., 2020; Greenberg et al., 2014; Agarwal et al., 2004; World Health Organization, 2004; Pakistan Multicentre Amoxycillin Short Course Therapy MASCOT Pneumonia Study Group, 2002; Lupisan et al., 1999; Sadruddin et al., 2019). Therefore, the strongest inference from this review applies to selected children with non-severe pneumonia rather than to all uncomplicated pediatric bacterial infections. Evidence for urinary tract infection, tonsillitis or pharyngotonsillitis, and other non-pneumonia infections was more limited and should be interpreted cautiously (Li et al., 2019; Montini et al., 2024; Roord et al., 1996).

The findings are consistent with previous evidence suggesting that shorter courses may be sufficient for selected children with non-severe community-acquired pneumonia (Haider et al., 2008; Williams et al., 2022; Bielicki et al., 2021; Pernica et al., 2021; Ginsburg et al., 2020; Kang, 2026; Li et al., 2022; Dutta et al., 2025). Our primary estimate is essentially identical to Li et al., who also restricted comparisons to the same oral antibiotic in childhood non-severe CAP and reported a pooled RR of 1.01 (95% CI, 0.92–1.11; I^2^ = 0%) (Li et al., 2022), and to Gao et al., who found no important difference between shorter and longer courses (Gao et al., 2023). Our review extends this work by also examining non-pneumonia infections (urinary tract infection and tonsillitis) in exploratory analyses, by reporting absolute as well as relative effects, and by separating same-antibiotic from different-antibiotic comparisons. Several contemporary randomized trials, including SCOUT-CAP, CAP-IT, SAFER, and Ginsburg et al., have shown that reduced antibiotic exposure can provide comparable clinical outcomes in carefully selected pediatric pneumonia populations (Williams et al., 2022; Bielicki et al., 2021; Pernica et al., 2021; Ginsburg et al., 2020; Kok et al., 2025). The SAFER trial, for example, concluded that 5 days of high-dose amoxicillin produced outcomes comparable to 10 days in previously healthy children with community-acquired pneumonia who did not require hospitalization, and it framed this interpretation within antimicrobial stewardship principles (Pernica et al., 2021). This wording is important: shorter treatment should be interpreted as comparable to, not superior to, longer treatment.

The duration-specific analyses provide a more clinically interpretable framework than a simple binary comparison of “short-course” versus “longer-course” therapy. A 5-day regimen may represent the longer arm in a 3-versus-5-day trial but the shorter arm in a 5-versus-10-day trial. In the present analysis, both the 3-versus-5-day and 5-versus-10-day comparisons were close to null, and there was no statistically significant subgroup difference by treatment-duration comparison. These findings support the interpretation that shorter therapy was not associated with higher treatment failure across the main duration contrasts studied, although heterogeneity remained in the broader analyses (Williams et al., 2022; Bielicki et al., 2021; Pernica et al., 2021; Ginsburg et al., 2020; Greenberg et al., 2014; Agarwal et al., 2004; World Health Organization, 2004; Pakistan Multicentre Amoxycillin Short Course Therapy MASCOT Pneumonia Study Group, 2002; Lupisan et al., 1999; Sadruddin et al., 2019; Montini et al., 2024).

The inclusion of absolute risk difference strengthens clinical interpretation. Relative measures alone may be difficult to apply when baseline risks differ across settings. In this review, the pooled RD was 0.001, indicating a very small absolute difference between short-course and longer-course therapy. This absolute-effect estimate supports the interpretation of comparable effectiveness and is clinically relevant because antibiotic-duration decisions require balancing comparable clinical effectiveness against potential benefits of shorter therapy, including reduced antibiotic exposure, improved adherence, lower cost, and reduced selection pressure for antimicrobial resistance (Barlam et al., 2016; Langdon et al., 2016; Spellberg, 2016; Llewelyn et al., 2017).

Country-income subgroup analysis showed no evidence that treatment effect differed between low- and middle-income country studies and high-income country studies. Low- and middle-income country trials contributed most participants, whereas high-income country trials contributed fewer but more recent comparisons. Heterogeneity was higher among low- and middle-income country studies, which may reflect differences in diagnostic criteria, follow-up systems, nutritional status, background disease severity, access to care, local resistance patterns, and thresholds for defining treatment failure. These contextual factors should be considered when applying the findings across healthcare settings (Ginsburg et al., 2020; Agarwal et al., 2004; World Health Organization, 2004; Pakistan Multicentre Amoxycillin Short Course Therapy MASCOT Pneumonia Study Group, 2002; Lupisan et al., 1999; Sadruddin et al., 2019).

Ancillary clinical outcome analyses also showed no statistically significant difference between short-course and longer-course therapy. However, these analyses were based on a small number of comparisons and should be considered supportive rather than definitive (Li et al., 2019). Outcomes beyond initial treatment failure, including relapses, recurrence, re-treatment, adverse events, microbiome effects, and antimicrobial resistance, were less consistently reported. This is important because the broader rationale for shorter therapy depends not only on comparable clinical effectiveness but also on reducing avoidable antibiotic exposure and its downstream consequences (Barlam et al., 2016; Langdon et al., 2016; Spellberg, 2016; Llewelyn et al., 2017; Xu et al., 2025).

Risk-of-bias assessment showed that trials varied in overall risk of bias, with 5 trials rated as low risk, 2 as having some concerns, and 7 as high risk (Sterne et al., 2019). Leave-one-out sensitivity analyses did not materially change the main interpretation, suggesting that the pooled finding was not driven by any single study. Nevertheless, in the broader and exploratory analyses, the higher statistical heterogeneity indicates that those pooled estimates should be interpreted as broad summaries of randomized evidence rather than as a uniform treatment effect applicable to every pediatric infection or treatment setting.

This review has several strengths. It focused on randomized clinical trials, included both relative and absolute effect measures, examined duration-specific and country-income subgroups, assessed risk of bias, and evaluated robustness using sensitivity analyses (Page et al., 2021; Sterne et al., 2019; DerSimonian and Laird, 1986; Egger et al., 1997). The analysis also aligns with the comparative-effectiveness framing used in many antibiotic-duration trials, where the clinical question is whether shorter treatment achieves comparable clinical outcomes with reduced antibiotic exposure, rather than whether it is superior to longer treatment (Williams et al., 2022; Bielicki et al., 2021; Pernica et al., 2021; Ginsburg et al., 2020).

Several limitations should be acknowledged. First, although the primary same-antibiotic pneumonia/lower respiratory tract infection analysis was statistically homogeneous (I^2^ = 0%), the broader and exploratory analyses showed substantial heterogeneity that limits the certainty and generalizability of those pooled estimates; we did not perform meta-regression because the primary analysis was homogeneous. Second, most included trials evaluated pneumonia, while evidence for other uncomplicated pediatric bacterial infections was sparse. The included children also spanned a wide age range, across which the microbiology and clinical course of childhood pneumonia differ, and pathogen-specific data were rarely reported, so the pooled estimate may obscure age- or pathogen-related differences in treatment response. Third, outcome definitions varied across trials, requiring harmonization of clinically related but non-identical unfavorable clinical endpoints. Fourth, some subgroup categories included few studies or single trials and should not be overinterpreted. Fifth, adverse events, recurrence, re-treatment, microbiome outcomes, and antimicrobial resistance outcomes were inconsistently reported. Finally, because the evidence base included different duration contrasts, infection types, and clinical settings, the findings should be applied using infection-specific clinical judgment rather than as a universal rule for shortening all pediatric antibiotic courses.

Overall, these findings support an infection-specific and duration-specific stewardship approach. Short-course oral antibiotic therapy was not associated with a statistically detectable increase in treatment failure compared with standard- or longer-course therapy for selected children with uncomplicated bacterial infections, particularly where syndrome-specific randomized evidence supports shorter regimens. Treatment duration should continue to account for infection type, disease severity, age, antibiotic choice, local resistance patterns, follow-up reliability, and guideline recommendations.

## Conclusion

In same-antibiotic pediatric pneumonia/LRTI trials, short-course oral antibiotic therapy was not associated with increased clinical treatment failure compared with standard- or longer-course therapy. Broader analyses across different antibiotic strategies and infection syndromes did not materially change the overall interpretation but showed substantial heterogeneity. These findings support cautious, infection-specific antibiotic duration reduction in non-severe, outpatient-managed pediatric pneumonia/LRTI treated with the antibiotics evaluated here, rather than broad generalization to all uncomplicated bacterial infections.

## Data Availability

The original contributions presented in the study are included in the article/Supplementary Material, further inquiries can be directed to the corresponding authors.
