# Supplementary material for: Short-course versus standard- or longer-course oral antibiotic therapy for pneumonia and lower respiratory tract infection in children: a systematic review and meta-analysis of randomized controlled trials
Source: Front Pharmacol. 2026 Oct 14;17:1877367. doi: 10.3389/fphar.2026.1877367 (PMC13713302; doi:10.3389/fphar.2026.1877367)
Supplement: Supplementary file 1 [file Supplementaryfile1.docx]

**Supplementary file**

**Figure S1. Primary risk-difference meta-analysis for treatment failure in same-antibiotic pediatric pneumonia/LRTI trials**

**Table S1. Leave-one-out analysis**

| **Excluded study** | **RD** | **95%CI** | **I^2^ (%)** | **tau^2^** | **Q_p** |
| --- | --- | --- | --- | --- | --- |
| MASCOT_2002 | 0.0004 | -0.012, 0.013 | 20 | 0.00007 | 0.27 |
| ISCAP_2004 | 0.0004 | -0.013, 0.014 | 20 | 0.00008 | 0.26 |
| Lupisan_1999 | 0.0019 | -0.010, 0.014 | 20 | 0.00006 | 0.28 |
| Greenberg_2014_3d | 0.0015 | -0.008, 0.011 | 0 | 0 | 0.95 |
| Ginsburg_2020 | -0.0014 | -0.016, 0.013 | 10 | 0.00007 | 0.31 |
| SAFER_2021 | 0.0016 | -0.010, 0.014 | 20 | 0.00006 | 0.27 |
| CAPIT_2021 | 0.0012 | -0.011, 0.014 | 20 | 0.00007 | 0.26 |
| SCOUTCAP_2022 | 0.0027 | -0.011, 0.016 | 20 | 0.00007 | 0.29 |
| Kartasasmita_2004 | 0.0029 | -0.009, 0.015 | 20 | 0.00004 | 0.32 |

**Table S2. Exploratory analysis**

| **Analysis** | **k** | **RR (95% CI)** | **RR I²** | **RD (95% CI)** | **RD I²** |
| --- | --- | --- | --- | --- | --- |
| Primary outcome | 9 | 1.01 (0.92 to 1.11) | 0.0% | 0.001 (−0.010 to 0.013) | 10.0% |
| Broad pneumonia/LRTI sensitivity | 12 | 0.94 (0.66 to 1.33) | 90% | −0.002 (−0.029 to 0.025) | 90% |
| All-infection exploratory analysis | 13 | 0.89 (0.63 to 1.25) | 90% | −0.007 (−0.033 to 0.019) | 90% |

K - No. pooled comparisons

**Table S3A. GRADE certainty assessment for the primary outcome**

| **Outcome** | **Study design** | **Comparisons** | **Participants** | **Risk of bias** | **Inconsistency** | **Indirectness** | **Imprecision** | **Publication bias** | **Final certainty** |
| --- | --- | --- | --- | --- | --- | --- | --- | --- | --- |
| Clinical treatment failure | RCTs | 9 | 10,883 | Serious | Not serious | Not serious | Not serious | Not serious* | ⊕⊕⊕◯ Moderate |

*Publication bias was not downgraded, but formal assessment was limited because fewer than 10 comparisons contributed to the primary analysis.

**Table S3B. GRADE summary of findings for the primary same-antibiotic pediatric pneumonia/LRTI analysis**

| **Outcome** | **Relative effect** | **Absolute effect** | **Heterogeneity** | **Certainty** | **Interpretation** |
| --- | --- | --- | --- | --- | --- |
| Clinical treatment failure | RR 1.01; 95% CI, 0.92–1.11 | RD 0.001; 95% CI, −0.010 to 0.013 | RR I² = 0.0%; RD I² = 10.0% | ⊕⊕⊕◯ Moderate | Little to no difference |

**Absolute interpretation:** 1 more treatment failure per 1,000 children, ranging from 10 fewer to 13 more per 1,000.

**Abbreviations:** CI, confidence interval; GRADE, Grading of Recommendations Assessment, Development and Evaluation; LRTI, lower respiratory tract infection; RCT, randomized controlled trial; RD, risk difference; RR, risk ratio.

**Figure S2. Funnel plot for the broad pneumonia/LRTI sensitivity analysis**

Funnel plot assessing small-study effects in the broad pneumonia/lower respiratory tract infection (LRTI) sensitivity analysis, which included same-antibiotic pneumonia/LRTI comparisons and additional different-antibiotic comparisons. Each point represents one randomized comparison. Study labels are omitted for visual clarity. No clear funnel-plot asymmetry was observed, and Egger’s regression test was not statistically significant (P = 0.247). Interpretation should remain cautious because the number of comparisons was limited and clinical heterogeneity was substantial.

**Figure S3. Funnel plot for the all-infection exploratory analysis**

Funnel plot assessing small-study effects in the all-infection exploratory treatment-failure analysis. This analysis included pneumonia/LRTI comparisons, different-antibiotic comparisons, and non-respiratory infection data. Each point represents one randomized comparison. Study labels are omitted for visual clarity. No clear funnel-plot asymmetry was observed, and Egger’s regression test was not statistically significant (P = 0.363). Because this analysis combined clinically heterogeneous infection syndromes and treatment strategies, the funnel plot was interpreted as exploratory only.

**Figure S4. Risk-of-bias traffic-light plot for included randomized trials**

**Figure S5. Overall risk-of-bias summary across included randomized trials**

**Figure S6. Broad pneumonia/LRTI sensitivity analysis of treatment failure (risk ratio). Random-effects meta-analysis of 12 comparisons the nine same-antibiotic pneumonia/LRTI trials plus three trials that differed in antibiotic class as well as duration (Sadruddin 2019; Awasthi 2008; Roord 1996). Pooled RR 0.94 (95% CI, 0.66–1.33) with substantial heterogeneity (I² = 93.2%). The wide interval and high heterogeneity reflect the inclusion of different-antibiotic comparisons; this analysis is interpreted as exploratory rather than confirmatory.**

**Table S4. Contribution of each included trial to the primary, broad sensitivity, and all-infection analyses**

| **Trial (comparison)** | **Condition** | **Same antibiotic** | **Primary analysis (k = 9)** | **Broad sensitivity (k = 12)** | **All-infection exploratory (k = 13)** |
| --- | --- | --- | --- | --- | --- |
| SCOUT-CAP 2022 | Pneumonia/LRTI | Yes | Included | Included | Included |
| CAP-IT 2021 | Pneumonia/LRTI | Yes | Included | Included | Included |
| SAFER 2021 | Pneumonia/LRTI | Yes | Included | Included | Included |
| Ginsburg 2020 | Pneumonia/LRTI | Yes | Included | Included | Included |
| Greenberg 2014, 3-day | Pneumonia/LRTI | Yes | Included | Included | Included |
| Greenberg 2014, 5-day | Pneumonia/LRTI | Yes | Risk difference only | Risk difference only | Risk difference only |
| ISCAP 2004 | Pneumonia/LRTI | Yes | Included | Included | Included |
| Kartasasmita 2004 | Pneumonia/LRTI | Yes | Included | Included | Included |
| MASCOT 2002 | Pneumonia/LRTI | Yes | Included | Included | Included |
| Lupisan 1999 | Pneumonia/LRTI | Yes | Included | Included | Included |
| Sadruddin 2019 | Pneumonia/LRTI | No | — | Included | Included |
| Awasthi 2008 | Pneumonia/LRTI | No | — | Included | Included |
| Roord 1996 | Pneumonia/LRTI | No | — | Included | Included |
| STOP 2024 | Urinary tract infection | Yes | — | — | Included |
| Li 2019 | Tonsillitis | No | — | — | — |

*Included denotes that the comparison contributed data to that pooled analysis. The Greenberg 2014 5-day stratum recorded no events in either arm; the risk ratio is not estimable for a double-zero comparison, so it contributes to risk-difference analyses only. Sadruddin 2019, Awasthi 2008 and Roord 1996 differed in antibiotic class as well as duration and were therefore excluded from the primary same-antibiotic analysis. STOP 2024 evaluated a non-respiratory infection and contributed only to the all-infection exploratory analysis. Li 2019 evaluated tonsillitis and contributed to the systematic review and risk-of-bias assessment only; it was not entered into the quantitative dataset. References [16-24] correspond to the nine comparisons of the primary analysis.*

**Table S5. Reconciliation of randomised participants (Table 1) with participants contributing to the primary pooled analysis (Figure 2)**

| **Trial** | **Randomised (Table 1)** | **Contributing to primary analysis (Figure 2)** | **Difference** | **Reason for difference** |
| --- | --- | --- | --- | --- |
| MASCOT 2002 | 2,000 | 2,000 | 0 | All randomised participants contributed outcome data |
| ISCAP 2004 | 2,188 | 2,188 | 0 | All randomised participants contributed outcome data |
| Lupisan 1999 | 303 | 303 | 0 | All randomised participants contributed outcome data |
| Greenberg 2014 | 140 | 27 | 113 | Only the 3-day stratum contributes to the risk-ratio analysis; the 5-day stratum (n = 91) recorded no events in either arm and is not estimable for the risk ratio |
| Ginsburg 2020 | 3,000 | 2,898 | 102 | Participants without evaluable treatment-failure outcome data |
| SAFER 2021 | 281 | 251 | 30 | Participants without evaluable treatment-failure outcome data |
| CAP-IT 2021 | 824 | 814 | 10 | Participants without evaluable treatment-failure outcome data |
| SCOUT-CAP 2022 | 385 | 380 | 5 | Participants without evaluable treatment-failure outcome data |
| Kartasasmita 2004 | 2,024 | 2,022 | 2 | Participants without evaluable treatment-failure outcome data |
| Total | 11,145 | 10,883 | 262 |  |

*Table 1 reports the total number of participants randomised in each trial. Figure 2 analyses only those participants who contributed evaluable treatment-failure outcome data to the pooled comparison, which is smaller in several trials. The two columns therefore answer different questions and are not expected to be identical.*
