# Supplementary material for: Short-course versus standard- or longer-course oral antibiotic therapy for pneumonia and lower respiratory tract infection in children: a systematic review and meta-analysis of randomized controlled trials
Source: Front Pharmacol. 2026 Oct 14;17:1877367. doi: 10.3389/fphar.2026.1877367 (PMC13713302; doi:10.3389/fphar.2026.1877367)
Supplement: Supplementary file 2 [file Supplementaryfile2.docx]

**Supplementary Appendix S1. Full search strategies**

*All searches were run from database inception to the date shown. Searches were limited to published English-language studies, consistent with the registered protocol. Concept labels are provided for readability; search terms are reproduced exactly as executed.*

**Table S1.1. Sources searched, dates, and records retrieved**

| **Source** | **Platform / interface** | **Date last searched** | **Records retrieved** |
| --- | --- | --- | --- |
| PubMed / MEDLINE | PubMed (pubmed.ncbi.nlm.nih.gov) | 1 May 2026 | — |
| Embase | Ovid | 1 May 2026 | — |
| MEDLINE | Ovid | 1 May 2026 | — |
| Cochrane CENTRAL | Cochrane Library | 1 May 2026 | — |
| Web of Science | Clarivate Web of Science Core Collection | 1 May 2026 | — |
| ClinicalTrials.gov | clinicaltrials.gov | 1 May 2026 | — |
| WHO ICTRP | trialsearch.who.int | 1 May 2026 | — |
| Citation searching | Reference lists of included trials and relevant reviews; forward-citation tracking | 1 May 2026 | — |
| **Total records identified** | All sources combined | 1 May 2026 | **1,352** |

*Records retrieved from individual sources were not retained; the total of 1,352 records reflects all sources combined before deduplication (1,083 duplicates removed; 269 records screened). Web of Science was searched in addition to the databases listed in the registered PROSPERO protocol (CENTRAL, Embase, MEDLINE, PubMed).*

**1. PubMed / MEDLINE**

| **Set** | **Concept** | **Search terms** |
| --- | --- | --- |
| #1 | Population (children) | child*[Title/Abstract] OR pediatric*[Title/Abstract] OR paediatric*[Title/Abstract] OR infant*[Title/Abstract] OR toddler*[Title/Abstract] OR adolescent*[Title/Abstract] OR "Child"[Mesh] OR "Infant"[Mesh] OR "Adolescent"[Mesh] |
| #2 | Antibiotics | antibiotic*[Title/Abstract] OR antibacterial*[Title/Abstract] OR anti-bacterial*[Title/Abstract] OR "Anti-Bacterial Agents"[Mesh] OR amoxicillin[Title/Abstract] OR amoxycillin[Title/Abstract] OR amoxicillin-clavulanate[Title/Abstract] OR co-amoxiclav[Title/Abstract] OR cotrimoxazole[Title/Abstract] OR co-trimoxazole[Title/Abstract] OR trimethoprim-sulfamethoxazole[Title/Abstract] OR azithromycin[Title/Abstract] OR erythromycin[Title/Abstract] OR cefaclor[Title/Abstract] OR cephalosporin*[Title/Abstract] OR beta-lactam*[Title/Abstract] |
| #3 | Treatment duration | "short course"[Title/Abstract] OR short-course[Title/Abstract] OR "short duration"[Title/Abstract] OR reduced-duration[Title/Abstract] OR "reduced duration"[Title/Abstract] OR "treatment duration"[Title/Abstract] OR "duration of therapy"[Title/Abstract] OR "antibiotic duration"[Title/Abstract] OR "standard course"[Title/Abstract] OR standard-course[Title/Abstract] OR "long course"[Title/Abstract] OR long-course[Title/Abstract] OR "3 day"[Title/Abstract] OR "3-day"[Title/Abstract] OR "three day"[Title/Abstract] OR "three-day"[Title/Abstract] OR "5 day"[Title/Abstract] OR "5-day"[Title/Abstract] OR "five day"[Title/Abstract] OR "five-day"[Title/Abstract] OR "7 day"[Title/Abstract] OR "7-day"[Title/Abstract] OR "seven day"[Title/Abstract] OR "seven-day"[Title/Abstract] OR "10 day"[Title/Abstract] OR "10-day"[Title/Abstract] OR "ten day"[Title/Abstract] OR "ten-day"[Title/Abstract] |
| #4 | Infections | pneumonia[Title/Abstract] OR "community-acquired pneumonia"[Title/Abstract] OR "lower respiratory tract infection"[Title/Abstract] OR LRTI[Title/Abstract] OR "Respiratory Tract Infections"[Mesh] OR "Pneumonia"[Mesh] OR "otitis media"[Title/Abstract] OR "Otitis Media"[Mesh] OR pharyngitis[Title/Abstract] OR tonsillitis[Title/Abstract] OR "Pharyngitis"[Mesh] OR "Tonsillitis"[Mesh] OR sinusitis[Title/Abstract] OR "Sinusitis"[Mesh] OR "urinary tract infection"[Title/Abstract] OR UTI[Title/Abstract] OR "Urinary Tract Infections"[Mesh] OR "skin infection"[Title/Abstract] OR "soft tissue infection"[Title/Abstract] OR "Skin Diseases, Bacterial"[Mesh] |
| #5 | Study design | randomized[Title/Abstract] OR randomised[Title/Abstract] OR randomly[Title/Abstract] OR trial[Title/Abstract] OR placebo[Title/Abstract] OR "Randomized Controlled Trial"[Publication Type] OR "Controlled Clinical Trial"[Publication Type] |
| #6 | Adult-only exclusion | adult*[Title/Abstract] NOT child*[Title/Abstract] |
| #7 | Combination | #1 AND #2 AND #3 AND #4 AND #5 NOT #6 |

**2. Embase via Ovid**

| **Set** | **Concept** | **Search terms** |
| --- | --- | --- |
| 1 | Population (children) | exp child/ OR exp infant/ OR exp adolescent/ OR child*.ti,ab. OR pediatric*.ti,ab. OR paediatric*.ti,ab. OR infant*.ti,ab. OR toddler*.ti,ab. OR adolescent*.ti,ab. |
| 2 | Antibiotics | exp antibiotic agent/ OR antibiotic*.ti,ab. OR antibacterial*.ti,ab. OR anti-bacterial*.ti,ab. OR amoxicillin.ti,ab. OR amoxycillin.ti,ab. OR amoxicillin-clavulanate.ti,ab. OR co-amoxiclav.ti,ab. OR cotrimoxazole.ti,ab. OR co-trimoxazole.ti,ab. OR trimethoprim-sulfamethoxazole.ti,ab. OR azithromycin.ti,ab. OR erythromycin.ti,ab. OR cefaclor.ti,ab. OR cephalosporin*.ti,ab. OR beta-lactam*.ti,ab. |
| 3 | Treatment duration | short-course.ti,ab. OR "short course".ti,ab. OR "short duration".ti,ab. OR reduced-duration.ti,ab. OR "reduced duration".ti,ab. OR "treatment duration".ti,ab. OR "duration of therapy".ti,ab. OR "antibiotic duration".ti,ab. OR standard-course.ti,ab. OR "standard course".ti,ab. OR long-course.ti,ab. OR "long course".ti,ab. OR "3 day".ti,ab. OR "3-day".ti,ab. OR "three day".ti,ab. OR "three-day".ti,ab. OR "5 day".ti,ab. OR "5-day".ti,ab. OR "five day".ti,ab. OR "five-day".ti,ab. OR "7 day".ti,ab. OR "7-day".ti,ab. OR "seven day".ti,ab. OR "seven-day".ti,ab. OR "10 day".ti,ab. OR "10-day".ti,ab. OR "ten day".ti,ab. OR "ten-day".ti,ab. |
| 4 | Infections | exp pneumonia/ OR exp respiratory tract infection/ OR pneumonia.ti,ab. OR "community-acquired pneumonia".ti,ab. OR "community acquired pneumonia".ti,ab. OR "lower respiratory tract infection".ti,ab. OR LRTI.ti,ab. OR exp otitis media/ OR "otitis media".ti,ab. OR exp pharyngitis/ OR pharyngitis.ti,ab. OR tonsillitis.ti,ab. OR exp sinusitis/ OR sinusitis.ti,ab. OR exp urinary tract infection/ OR "urinary tract infection".ti,ab. OR UTI.ti,ab. OR exp skin infection/ OR "skin infection".ti,ab. OR "soft tissue infection".ti,ab. |
| 5 | Study design | randomized controlled trial/ OR controlled clinical trial/ OR random*.ti,ab. OR randomised.ti,ab. OR randomized.ti,ab. OR trial.ti,ab. OR placebo.ti,ab. |
| 6 | Combination | 1 AND 2 AND 3 AND 4 AND 5 |
| 7 | Limit — humans | limit 6 to human |
| 8 | Limit — language | limit 7 to English language |
| 9 | Limit — publication type | limit 8 to article |

**3. Ovid MEDLINE**

| **Set** | **Concept** | **Search terms** |
| --- | --- | --- |
| 1 | Population (children) | exp Child/ OR exp Infant/ OR exp Adolescent/ OR child*.ti,ab. OR pediatric*.ti,ab. OR paediatric*.ti,ab. OR infant*.ti,ab. OR toddler*.ti,ab. OR adolescent*.ti,ab. |
| 2 | Antibiotics | exp Anti-Bacterial Agents/ OR antibiotic*.ti,ab. OR antibacterial*.ti,ab. OR anti-bacterial*.ti,ab. OR amoxicillin.ti,ab. OR amoxycillin.ti,ab. OR amoxicillin-clavulanate.ti,ab. OR co-amoxiclav.ti,ab. OR cotrimoxazole.ti,ab. OR co-trimoxazole.ti,ab. OR trimethoprim-sulfamethoxazole.ti,ab. OR azithromycin.ti,ab. OR erythromycin.ti,ab. OR cefaclor.ti,ab. OR cephalosporin*.ti,ab. OR beta-lactam*.ti,ab. |
| 3 | Treatment duration | short-course.ti,ab. OR "short course".ti,ab. OR "short duration".ti,ab. OR reduced-duration.ti,ab. OR "reduced duration".ti,ab. OR "treatment duration".ti,ab. OR "duration of therapy".ti,ab. OR "antibiotic duration".ti,ab. OR standard-course.ti,ab. OR "standard course".ti,ab. OR long-course.ti,ab. OR "long course".ti,ab. OR "3 day".ti,ab. OR "3-day".ti,ab. OR "three day".ti,ab. OR "three-day".ti,ab. OR "5 day".ti,ab. OR "5-day".ti,ab. OR "five day".ti,ab. OR "five-day".ti,ab. OR "7 day".ti,ab. OR "7-day".ti,ab. OR "seven day".ti,ab. OR "seven-day".ti,ab. OR "10 day".ti,ab. OR "10-day".ti,ab. OR "ten day".ti,ab. OR "ten-day".ti,ab. |
| 4 | Infections | exp Pneumonia/ OR exp Respiratory Tract Infections/ OR pneumonia.ti,ab. OR "community-acquired pneumonia".ti,ab. OR "community acquired pneumonia".ti,ab. OR "lower respiratory tract infection".ti,ab. OR LRTI.ti,ab. OR exp Otitis Media/ OR "otitis media".ti,ab. OR exp Pharyngitis/ OR pharyngitis.ti,ab. OR tonsillitis.ti,ab. OR exp Sinusitis/ OR sinusitis.ti,ab. OR exp Urinary Tract Infections/ OR "urinary tract infection".ti,ab. OR UTI.ti,ab. OR exp Skin Diseases, Bacterial/ OR "skin infection".ti,ab. OR "soft tissue infection".ti,ab. |
| 5 | Study design | randomized controlled trial.pt. OR controlled clinical trial.pt. OR random*.ti,ab. OR randomised.ti,ab. OR randomized.ti,ab. OR trial.ti,ab. OR placebo.ti,ab. |
| 6 | Combination | 1 AND 2 AND 3 AND 4 AND 5 |
| 7 | Limit — humans | limit 6 to humans |
| 8 | Limit — language | limit 7 to English language |

**4. Cochrane CENTRAL**

| **Set** | **Concept** | **Search terms** |
| --- | --- | --- |
| #1 | Population (MeSH) | MeSH descriptor: [Child] explode all trees |
| #2 | Population (MeSH) | MeSH descriptor: [Infant] explode all trees |
| #3 | Population (MeSH) | MeSH descriptor: [Adolescent] explode all trees |
| #4 | Population (free text) | child* OR pediatric* OR paediatric* OR infant* OR toddler* OR adolescent* |
| #5 | Population combined | #1 OR #2 OR #3 OR #4 |
| #6 | Antibiotics (MeSH) | MeSH descriptor: [Anti-Bacterial Agents] explode all trees |
| #7 | Antibiotics (free text) | antibiotic* OR antibacterial* OR anti-bacterial* OR amoxicillin OR amoxycillin OR amoxicillin-clavulanate OR co-amoxiclav OR cotrimoxazole OR co-trimoxazole OR trimethoprim-sulfamethoxazole OR azithromycin OR erythromycin OR cefaclor OR cephalosporin* OR beta-lactam* |
| #8 | Antibiotics combined | #6 OR #7 |
| #9 | Treatment duration | "short course" OR short-course OR "short duration" OR reduced-duration OR "reduced duration" OR "treatment duration" OR "duration of therapy" OR "antibiotic duration" OR "standard course" OR standard-course OR "long course" OR long-course OR "3 day" OR "3-day" OR "three day" OR "three-day" OR "5 day" OR "5-day" OR "five day" OR "five-day" OR "7 day" OR "7-day" OR "seven day" OR "seven-day" OR "10 day" OR "10-day" OR "ten day" OR "ten-day" |
| #10 | Infections (MeSH) | MeSH descriptor: [Pneumonia] explode all trees |
| #11 | Infections (MeSH) | MeSH descriptor: [Respiratory Tract Infections] explode all trees |
| #12 | Infections (MeSH) | MeSH descriptor: [Otitis Media] explode all trees |
| #13 | Infections (MeSH) | MeSH descriptor: [Pharyngitis] explode all trees |
| #14 | Infections (MeSH) | MeSH descriptor: [Tonsillitis] explode all trees |
| #15 | Infections (MeSH) | MeSH descriptor: [Sinusitis] explode all trees |
| #16 | Infections (MeSH) | MeSH descriptor: [Urinary Tract Infections] explode all trees |
| #17 | Infections (MeSH) | MeSH descriptor: [Skin Diseases, Bacterial] explode all trees |
| #18 | Infections (free text) | pneumonia OR "community-acquired pneumonia" OR "community acquired pneumonia" OR "lower respiratory tract infection" OR LRTI OR "otitis media" OR pharyngitis OR tonsillitis OR sinusitis OR "urinary tract infection" OR UTI OR "skin infection" OR "soft tissue infection" |
| #19 | Infections combined | #10 OR #11 OR #12 OR #13 OR #14 OR #15 OR #16 OR #17 OR #18 |
| #20 | Combination | #5 AND #8 AND #9 AND #19 |

**5. Web of Science**

| **Set** | **Concept** | **Search terms** |
| --- | --- | --- |
| #1 | Population (children) | TS=(child* OR pediatric* OR paediatric* OR infant* OR toddler* OR adolescent*) |
| #2 | Antibiotics | TS=(antibiotic* OR antibacterial* OR anti-bacterial* OR amoxicillin OR amoxycillin OR amoxicillin-clavulanate OR co-amoxiclav OR cotrimoxazole OR co-trimoxazole OR trimethoprim-sulfamethoxazole OR azithromycin OR erythromycin OR cefaclor OR cephalosporin* OR beta-lactam*) |
| #3 | Treatment duration | TS=("short course" OR short-course OR "short duration" OR reduced-duration OR "reduced duration" OR "treatment duration" OR "duration of therapy" OR "antibiotic duration" OR "standard course" OR standard-course OR "long course" OR long-course OR "3 day" OR "3-day" OR "three day" OR "three-day" OR "5 day" OR "5-day" OR "five day" OR "five-day" OR "7 day" OR "7-day" OR "seven day" OR "seven-day" OR "10 day" OR "10-day" OR "ten day" OR "ten-day") |
| #4 | Infections | TS=(pneumonia OR "community-acquired pneumonia" OR "community acquired pneumonia" OR "lower respiratory tract infection" OR LRTI OR "otitis media" OR pharyngitis OR tonsillitis OR sinusitis OR "urinary tract infection" OR UTI OR "skin infection" OR "soft tissue infection") |
| #5 | Study design | TS=(random* OR randomized OR randomised OR trial OR placebo) |
| #6 | Combination | #1 AND #2 AND #3 AND #4 AND #5 |

*Abbreviations: exp, exploded subject heading; LRTI, lower respiratory tract infection; MeSH, Medical Subject Headings; .pt., publication type; .ti,ab., title/abstract field; TS, topic search; UTI, urinary tract infection.*
